# Hard-Diet Feeding Recovers Neurogenesis in the Subventricular Zone and Olfactory Functions of Mice Impaired by Soft-Diet Feeding

**DOI:** 10.1371/journal.pone.0097309

**Published:** 2014-05-09

**Authors:** Chizuru Utsugi, Sadaharu Miyazono, Kazumi Osada, Hitoshi Sasajima, Tomohiro Noguchi, Mitsuyoshi Matsuda, Makoto Kashiwayanagi

**Affiliations:** 1 Department of Oral and Maxillofacial Surgery, Asahikawa Medical University, Asahikawa, Japan; 2 Department of Sensory Physiology, Asahikawa Medical University, Asahikawa, Japan; 3 Department of Oral Biology, School of Dentistry, Health Sciences University of Hokkaido, Tohbetu, Japan; University of Barcelona, Parc Cientific de Barcelona and CIBERNED (ISCIII), Spain

## Abstract

The subventricular zone (SVZ) generates an immense number of neurons even during adulthood. These neurons migrate to the olfactory bulb (OB) and differentiate into granule cells and periglomerular cells. The information broadcast by general odorants is received by the olfactory sensory neurons and transmitted to the OB. Recent studies have shown that a reduction of mastication impairs both neurogenesis in the hippocampus and brain functions. To examine these effects, we first measured the difference in Fos-immunoreactivity (Fos-ir) at the principal sensory trigeminal nucleus (Pr5), which receives intraoral touch information via the trigeminal nerve, when female adult mice ingested a hard or soft diet to explore whether soft-diet feeding could mimic impaired mastication. Ingestion of a hard diet induced greater expression of Fos-ir cells at the Pr5 than did a soft diet or no diet. Bromodeoxyuridine-immunoreactive (BrdU-ir) structures in sagittal sections of the SVZ and in the OB of mice fed a soft or hard diet were studied to explore the effects of changes in mastication on newly generated neurons. After 1 month, the density of BrdU-ir cells in the SVZ and OB was lower in the soft-diet-fed mice than in the hard-diet-fed mice. The odor preferences of individual female mice to butyric acid were tested in a Y-maze apparatus. Avoidance of butyric acid was reduced by the soft-diet feeding. We then explored the effects of the hard-diet feeding on olfactory functions and neurogenesis in the SVZ of mice impaired by soft-diet feeding. At 3 months of hard-diet feeding, avoidance of butyric acid was reversed and responses to odors and neurogenesis were recovered in the SVZ. The present results suggest that feeding with a hard diet improves neurogenesis in the SVZ, which in turn enhances olfactory function at the OB.

## Introduction

A causal relationship between mastication and brain function has been observed in humans and animals [1]. Chewing ability correlates with cognitive impairment in elderly persons [2]. The Nun study, a longitudinal study of aging and Alzheimer's disease, indicated that participants with the fewest teeth had the highest prevalence and risk of incidence of dementia [3], suggesting the significance of mastication on brain function in humans [4]. Extraction of all molars of rats or shortening of the upper molars impairs spatial memory [5], [6]. Mastication is also impaired by offering animals only a soft diet (powdered food) [7], and thus performance on tests of working memory is lower in soft-diet-fed mice than hard-diet-fed mice [7]. One explanation for this may be that reduced sensory input influences neurogenesis [1].

Neurogenesis occurs in the forebrain subventricular zone (SVZ) throughout life. An immense number of neurons generated in the SVZ migrate via the rostral migratory stream (RMS) to the olfactory bulb (OB) [8]. Morphological and electrophysiological studies have shown that these cells differentiate into granule cells and periglomerular cells in the OB [8]–[10]. General odor information received by olfactory sensory neurons at the olfactory sensory epithelium is transmitted to the OB. Newly generated neurons at the SVZ play important roles in odor discrimination [11] and odor memory [12] in the OB.

New neurons are generated to ensure that the control of behavior by the mature nervous system is flexible under physiological changes [13] such as pregnancy and aging [14]–[17]. The SVZ's ability to generate neurons is lower in aged mice than young ones [16]. Aging in humans and mice impairs smell identification of general odors, olfactory discrimination learning, fine olfactory discrimination, and sensitivity to general odors [17]–[19]. These results provide a hypothesis that neurons migrated from the SVZ play a role in the levels of olfactory functions in aged animals. Reduction of neurogenesis by impaired mastication has been observed at the hippocampus in mice [20], [21]. Therefore, it is possible that impaired mastication also affects olfactory functions via changes in neurogenesis at the SVZ. However, it is not certain that a change in masticatory ability affects neurogenesis at the SVZ or the olfactory functions. In the present study, we compared 5-bromo-2′-deoxyuridine-immunoreactive (BrdU-ir) cells in the SVZ and OB of adult mice fed a soft diet. In addition, we measured the magnitude of avoidant behaviors in response to butyric acid of the soft-diet-fed mice to explore the physiological significance of mastication on the olfactory function. Finally, we explored the effects of the hard-diet feeding on the olfactory functions and neurogenesis at the SVZ of mice impaired by the soft-diet feeding.

## Results

### Soft-diet feeding reduced neurogenesis at the SVZ

**Figure 1:**
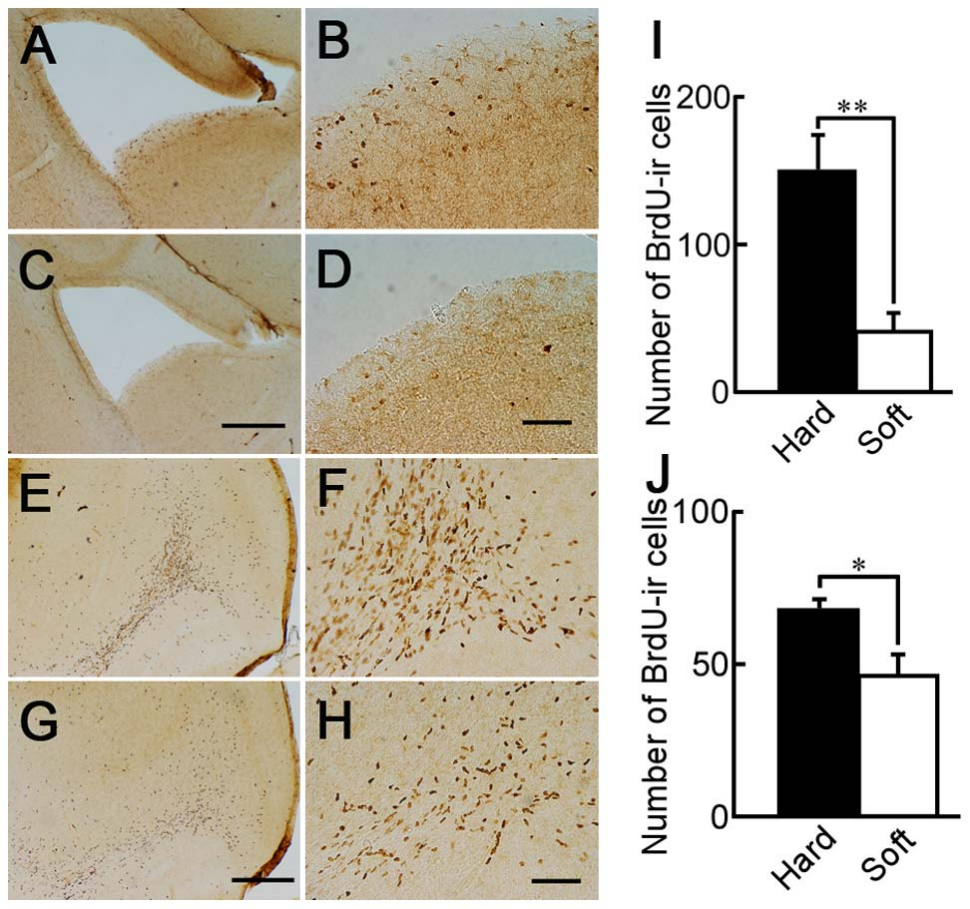
BrdU-ir cells in the SVZ and OB of mice fed a hard or soft diet. Sagittal sections of the SVZ (A–D) and OB (E–H) of mice fed a hard diet (A, B, E and F) or soft diet (C, D, G and H) for 1 month. Scale bar: 500 µm (C and G), 200 µm (D and H); I: the numbers of BrdU-ir cells at 600 µm thickness from Figure 108 of the mouse atlas (lateral 0.84 mm) of the SVZ to the lateral side of mice fed the hard diet (black column; n = 3) or soft diet (white column; n = 4). J: the number of BrdU-ir cells at 300 µm thickness from the section from Figure 108 of the mouse atlas (lateral 0.84 mm) of the OB to the lateral side of mice fed the hard diet (black column; n = 5) or soft diet (white column; n = 5). *: p<0.05; **: p<0.01.

Neurogenesis occurs robustly in the hippocampus and SVZ even in adulthood [13], [22]. In the present study, as shown in Figure 1A and B, there were many BrdU-ir cells in the SVZ of mice fed the normal hard diet. However, there were fewer BrdU-ir cells in the SVZ of mice fed the soft diet (powdered food) for 1 month (Figure 1C and D). Newly generated cells at the SVZ migrate to the OB via the RMS [8]. Many BrdU-ir cells were observed in the OB of mice fed the normal hard diet (Figure 1E and F), however, fewer BrdU-ir cells were observed in the OB of mice fed the soft diet for 1 month (Figure 1G and H). The data from each group were cast into a two-factor ANOVA as follows: diet (hard or soft) and regions (SVZ or OB). This analysis revealed a main effect of diet (F(1, 13) = 33.38, p<0.0001). An interaction between diet and regions were also found to be significant (F(1, 13) = 14.989, p<0.005). Fisher's PLSD post-hoc testing indicated that the number of BrdU-ir cells in hard diet fed mice was higher than that in soft diet fed mice (p<0.05). The number of BrdU-ir cells at the SVZ in mice fed the hard diet was higher than that of mice fed the soft diet (p<0.01), suggesting that a powdered diet led to a decrease in neurogenesis activity at the SVZ (Figure 1I). This would suggest that fewer cells migrated from the SVZ to the OB in the soft-diet-fed mice than in the hard diet-fed mice. Figure 1J shows the quantity of BrdU-ir cells at the OB of mice fed the soft or hard diet for 1 month. The numbers of BrdU-ir cells at the granule cell layer of the OB of mice fed the soft diet were lower than those of mice fed the hard diet for 1 month. The density of BrdU-ir cells in mice fed the soft diet was lower than that of mice fed the hard diet (p<0.05).

The dentate gyrus (DG) of the hippocampus is also a structure that exhibits neurogenesis throughout adulthood (for review see Aimone and Gage [23]). In the DG, in agreement with the results of a previous study [20], we found that the numbers of BrdU-ir cells of mice fed the soft diet for 3 months were lower than those of mice fed the hard diet for 3 months (p<0.05; Figure S1F). At 1 month of feeding, the numbers of BrdU-ir cells were almost the same between mice given a soft diet and those given a hard diet (Figure S1E). These results suggest that, in the soft-diet-fed mice, neurogenesis decreased in the SVZ before the DG.

### Soft-diet feeding impaired and hard-diet feeding recovered olfactory functions

**Figure 2:**
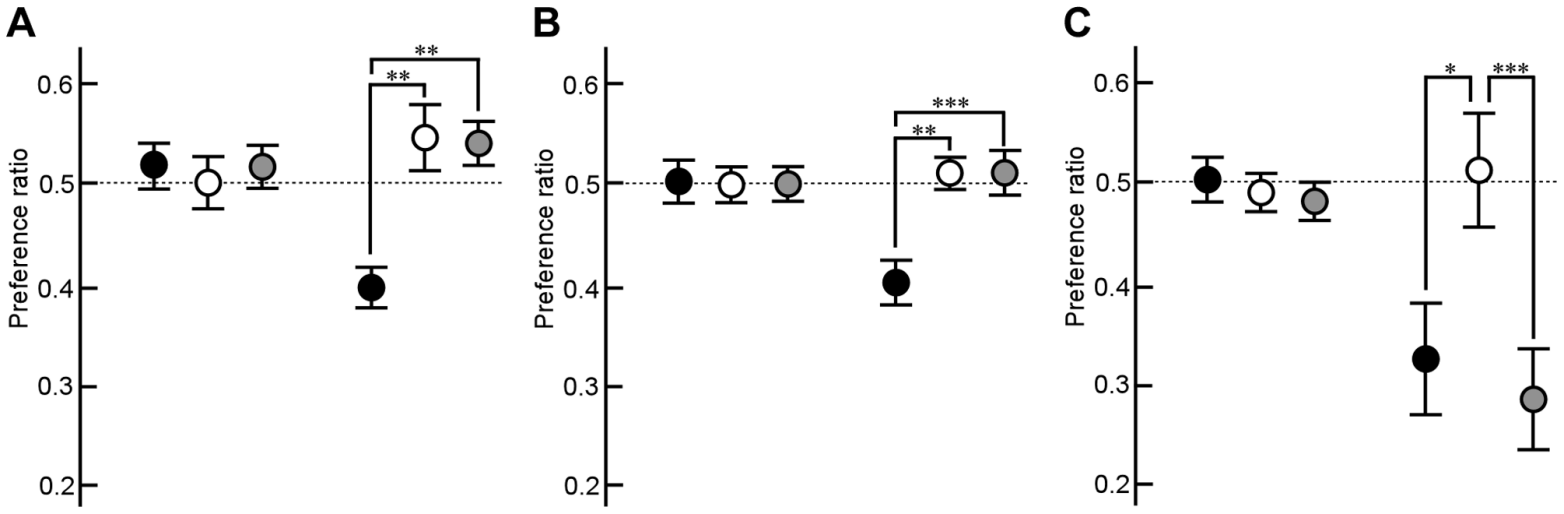
Avoidance of butyric acid in mice fed a hard diet, soft diet or hard diet after soft diet. Preferences were determined during a 4-min period in a Y-maze odor preference apparatus. Preference ratios between water placed on both sides of the Y-arm are shown in the three circles at the left in each figure (A, B, and C). When water was placed at the end of each Y-arm, the preference ratio of the mice fed the hard diet continuously, the soft diet continuously, the hard diet for 1 month after soft-diet feeding, or the hard diet for 3 months after soft-diet feeding was nearly 0.5. A: Preference ratios for 50% butyric acid in mice fed a hard diet (closed circles, n = 15) or soft diet (open circles and gray circles, n = 15 and n = 15, respectively). B: Preference ratios for 50% butyric acid in mice fed only a hard diet (closed circles, n = 15), only a soft diet (open circles, n = 15) or a hard diet after a soft diet (gray circles, n = 15) for 1 month. C: Preference ratios for 50% butyric acid in mice fed only a hard diet (closed circles, n = 10), only a soft diet (open circles, n = 10) and a hard diet after a soft diet (gray circles, n = 9) for 3 month. *: p<0.001; **: p<0.0005; ***: p<0.0001.

To explore the effects of a decrease in BrdU-ir cells in the OB of the soft-diet-fed mice on olfactory functions, we examined the magnitude of preference for 50% butyric acid using a Plexiglas Y-maze preference apparatus (Figure 2A). The data from each group were cast into a two-factor ANOVA as follows: hard or soft-diet feeding and preference between water and water or water and 50% butyric acid. The main effect of diet was found to be significant (F(1, 86) = 14.885, p<0.005). Fisher's PLSD post-hoc testing indicated that application of 50% butyric acid to mice fed the hard diet induced significant avoidance (p<0.005), while that to mice fed the soft diet did not. This suggested that mice fed the soft diet cannot recognize the odor of 50% butyric acid as undesirable.

Then, mice fed the soft diet were switched to the hard one for 1 or 3 months to explore the reversibility of the soft-diet-induced reduction of avoidance to the odor of 50% butyric acid. The data from each group were cast into a three-factor ANOVA as follows: hard-diet, soft-diet, or hard-diet after soft diet feeding; the period of hard-diet feeding; and stimulation. The main effects of diet, periods and stimulation were found to be significant (F(2, 136) = 10.288, p<0.0001; F(1, 136) = 22.897, p<0.0001; and F(1, 136) = 32.905, p<0.0001, respectively). The analysis also revealed a significant interaction between diets and periods, diet and stimulation, and period and stimulation (F(2, 136) = 8.191, p<0.0005; F(2, 136) = 11.203, p<0.0001; and F(1, 136) = 16.023; p<0.0005, respectively). In addition, the analysis revealed a significant interaction between diets, periods and simulation (F(2, 136) = 5.863, p<0.005). Fisher's PLSD post-hoc testing indicated that mice previously fed the soft diet for 1 month also did not show avoidance of 50% butyric acid after the hard-diet feeding for 1 month (p = 0.4999; Figure 2B). However, mice previously fed the soft diet for 1 month showed significant avoidance of 50% butyric acid after the hard-diet feeding for 3 months (p<0.0005), while mice continuously fed the soft diet did not (p = 0.8586; Figure 2C). These results suggest that the olfactory function involved in avoidance of 50% butyric acid, which was impaired by the soft-diet feeding, was nonetheless completely recovered by the hard-diet feeding for 3 months.

### Recovery of expression of Fos-ir cells at the OB and piriform cortex after hard-diet feeding

**Figure 3:**
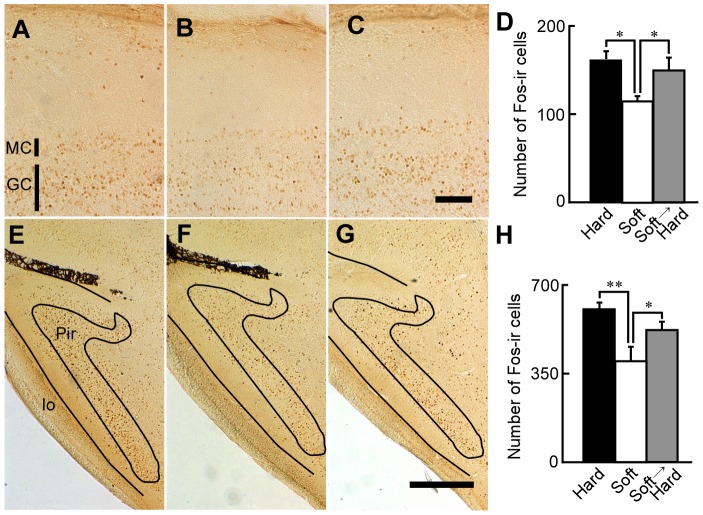
Fos-ir cells after exposure to urinary odor in the OB and Pir. Sagittal sections of the OB and Pir after exposure of mice fed only a hard diet (A and E), only a soft diet (B and F), or a hard diet for 3 months after a soft diet for 1 month (C and G) to urinary odor, respectively. Scale bar: 200 µm (C), 500 µm (G). D: The numbers of Fos-ir cells in the mitral cell layer in 500 µm thickness from Figure 108 of the mouse atlas (lateral 0.84 mm) of the OB to the lateral side of mice fed only the hard diet (black column; n = 5), only the soft diet (white column; n = 5), or the hard diet after the soft diet (gray column; n = 5). H: The number of Fos-ir cells in 400 µm thickness from Figure 114 of the mouse atlas (lateral 1.56 mm) of the Pir of mice fed only the hard diet (black column; n = 5), only the soft diet (white column; n = 5), or the hard diet after the soft diet (gray column; n = 5). *: p<0.05; **: p<0.005.

Next, we explored the effect of hard-diet feeding for 3 months on responses to odors at the OB and piriform cortex (Pir) of mice fed a soft diet for 1 month. Exposure to urinary odor excreted by males induced Fos-immunoreactivity (Fos-ir), which is correlated with cellular activity, in various cells of the OB of mice fed the hard diet (Figure 3A). In contrast, Fos-immunoreactivity at the OB of mice fed the soft diet was low (Figure 3B). In mice fed the hard diet for 3 months after the soft diet for 1 month, exposure to the urinary odor induced levels of Fos-ir cells at the OB that were similar to the levels in mice fed only the hard diet (Figure 3C). Figure 3D shows the number of Fos-ir cells at the mitral cell layer of the OB after exposure to urinary odor. The data from each group were cast into a one-factor ANOVA as follows: hard, soft, or hard after soft diet. One factor ANOVA revealed the main effect of diet (F(2, 12) = 4.78, p<0.05). Fisher's PLSD post-hoc testing indicated that the mitral cell layer of the OB in mice fed the hard diet for 3 months after the soft diet for 1 month, had more Fos-ir cells than the mice fed the soft diet alone (p<0.05). The hard-diet feeding also recovered odor responses at the Pir (Figure 3E-H). One-factor ANOVA revealed a main effect between diets (F(2, 12) = 6.63, p<0.05). Fisher's PLSD post-hoc testing indicated that the Pir in mice fed the hard diet for 3 months after the soft diet for 1 month had more Fos-ir cells than the Pir in mice fed the soft diet alone (p<0.05).

### Recovery of neurogenesis at the SVZ by hard-diet feeding

**Figure 4:**
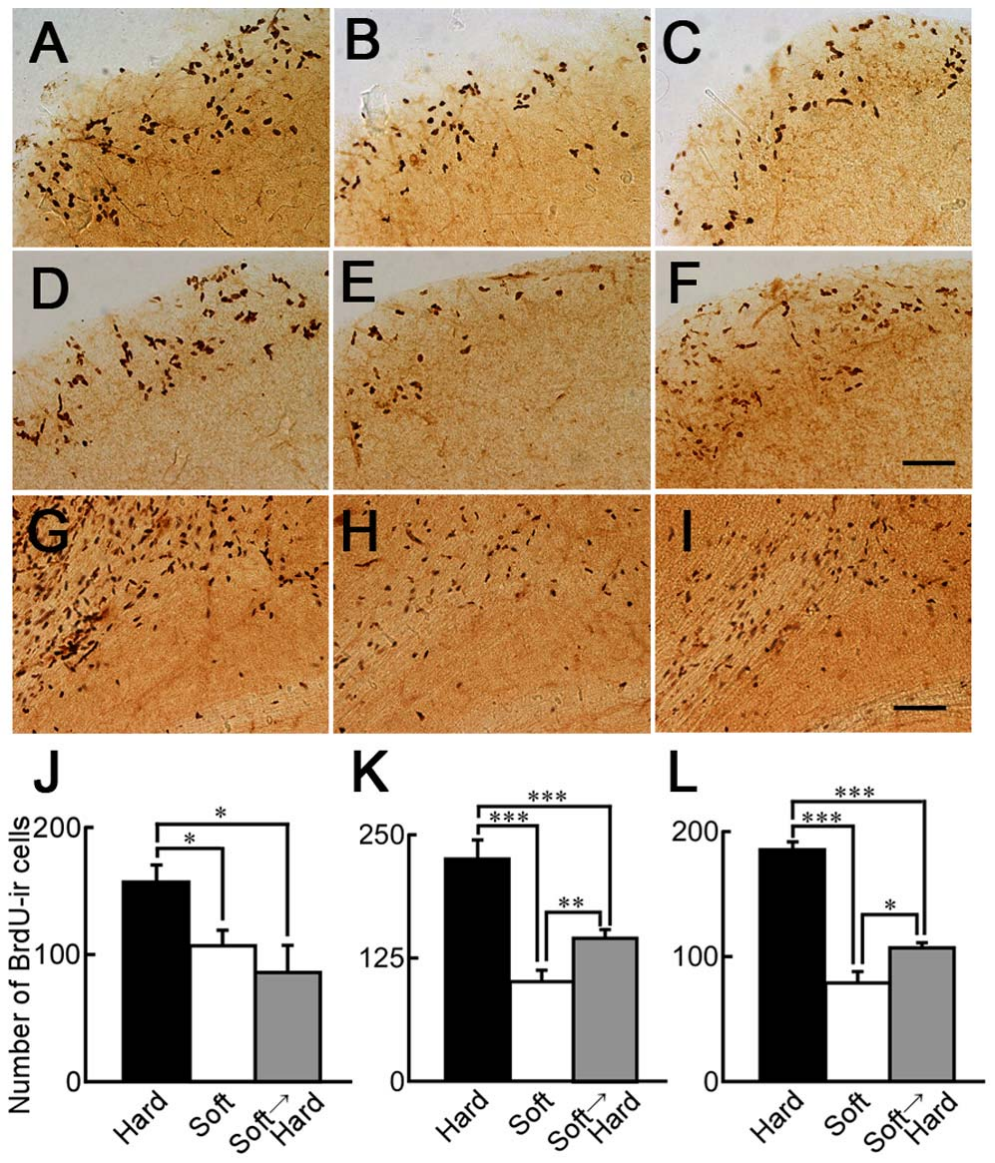
BrdU-ir cells in the SVZ of mice fed a hard diet for 1 or 3 months after being fed a soft diet. Sagittal sections of the SVZ of mice fed only a hard diet (A and D), only a soft diet (B and E), or a hard diet for 1 (C) or 3 months (F) after a soft diet for 1 month. Sagittal sections of the OB of mice fed only a hard diet (G), only a soft diet (H), and a hard diet for 3 months after a soft diet for 1 month (I). Scale bar: 200 µm (F and I); J and K: The numbers of BrdU-ir cells in 600 µm thickness from Figure 108 of the mouse atlas (lateral 0.84 mm) of the SVZ to the lateral side of mice fed only the hard diet (black column), only the soft diet (white column), or the hard diet for 1 month (J) or 3 months (K) after the soft diet (gray column). n = 4 without white column in K, n = 5 for white column in K. L: The number of BrdU-ir cells in 300 µm thickness from Figure 108 of the mouse atlas (lateral 0.84 mm) of the OB to the lateral side of mice fed only the hard diet (black column; n = 4), only the soft diet (white column; n = 5), or the hard diet for 3 months after the soft diet for 1 month (gray column; n = 4). *: p<0.05; **: p<0.01; ***: p<0.0001.

We next explored the effect of hard-diet feeding on neurogenesis at the SVZ and the number of newly generated cells at the OB in mice fed the soft diet for 1 month. Figure 4 shows BrdU-ir cells at the SVZ of mice fed only the hard diet, only the soft diet, or the hard diet for 1 or 3 months after the soft diet for 1 month. The expression of BrdU immunoreactivity at the SVZ of mice fed only the soft diet (B) was similar to that of mice fed the hard diet for 1 month after the soft diet for 1 month (C). However, the expression of the BrdU immunoreactivity of mice fed the hard diet for 3 months after the soft diet for 1 month (F) was higher than that of mice fed only the soft diet (E). At the SVZ, the data from each group were cast into a two-factor ANOVA as follows: hard, soft, or hard after soft diet and the period of hard-diet feeding. The main effect of diet was found to be significant (F (2, 19) = 28.928, p<0.0001). In mice given 1 month of a hard diet after 1 month of a soft diet, the number of BrdU-ir cells at the SVZ was similar to that of mice fed only the soft diet (Figure 4J). The number of BrdU-ir cells in mice given 3 months of a hard diet after 1 month of a soft diet was still smaller than that of mice fed only the hard diet (Figure 4K; p<0.0001), but it was significantly larger than that of mice fed only the soft diet (p<0.05). The number of BrdU-immunoreactive signals surrounded with doublecortin (DCX)-immunoreactive signals at the SVZ of mice fed a hard-diet after feeding with a soft-diet was higher than that of mice fed a soft-diet alone (Figure S2), suggesting that the reduced neurogenesis by soft-diet feeding was recovered by hard-diet feeding for 3 months.

Figure 4G-I shows the quantities of BrdU-ir cells at the granule cell layer of the OB of mice fed only the hard diet (G), only the soft diet (H), and the hard diet for 3 months after the soft diet (I). The data from each group were cast into a two-factor ANOVA as follows: diet (hard or soft) and regions (SVZ or OB). This analysis revealed a main effect of diet (F(2, 20) = 117.147, p<0.0001). Fisher's PLSD post-hoc testing indicated that the number of BrdU-ir cells in mice fed only the hard diet (black column) was higher than those in mice only the soft diet, or the hard diet for 3 months after the soft diet (p<0.0001). One-factor ANOVA revealed the main effect between diets (F(2, 10) = 62.751, p<0.001). Fisher's PLSD post-hoc testing indicated that the number of BrdU-ir cells at the OB of mice fed the hard diet for 3 months after the soft diet for 1 month was larger than that of mice fed only the soft diet (p<0.05).

### Expression of Fos-ir cells in the mouse brain after ingestion of hard or soft diet

**Figure 5:**
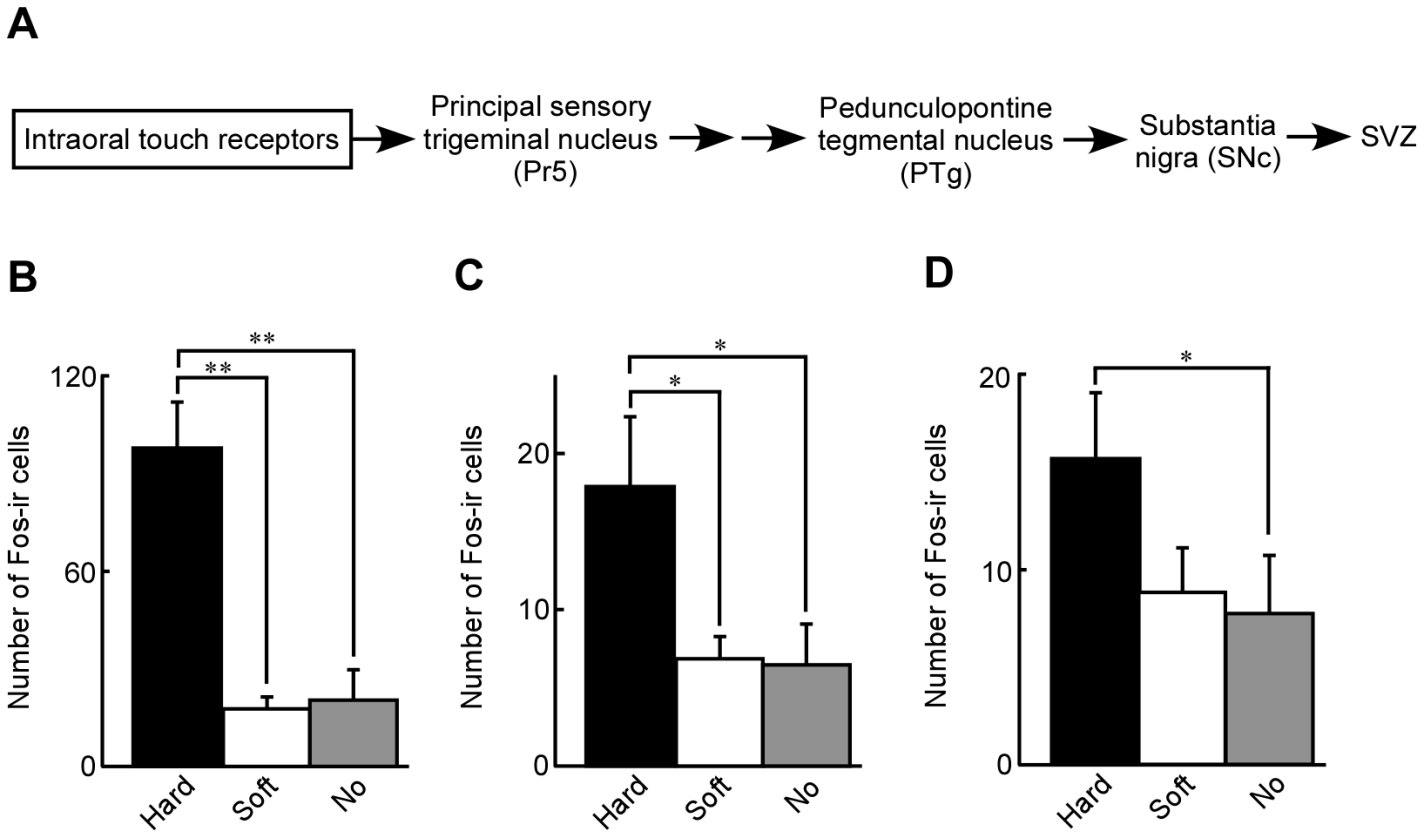
Effects of a hard or soft diet on the expression of Fos-ir cells in Pr5, PTg and SNc. A: A schematic transmission pathway of oral sensation from the mouth to the SVZ. The principal sensory trigeminal nucleus (Pr5), which receive intraoral touch information via the trigeminal nerve, transmits to the pedunculopontine tegmental nucleus (PTg) via the thalamus [24], somatosensory cortex [25], and motor cortex [26]. Neurons of the PTg innervate to the substantia nigra pars compacta (SNc) [27], [28], [47], in which dopaminergic neurons innervate to the SVZ [29], [30]. The number of Fos-ir cells in 100 µm thickness in Figure 115 of the mouse atlas (lateral 1.68 mm) of the Pr5 (B), 100 µm thickness in Figure 111 of the mouse atlas (lateral 1.20 mm) of PTg (C), and 200 µm thickness from Figure 106 of the mouse atlas (lateral 0.60 mm) of SNc (D) to the lateral side of mice after the ingestion of a hard diet (black column), soft diet fed (white column) or no diet (gray column). n = 4 (white and gray column in B). n = 5 (C). n = 10 (black and gray column in D). n = 11 (white column in D). *: p<0.05; **: p<0.005.

It is not clear exactly how the hard and soft diets induced different somatosensory sensations along the neural pathway from the mouths to the brains of mice. We therefore explored the difference in Fos-immunoreactivities at the principal sensory trigeminal nucleus (Pr5), which receives intraoral touch information via the trigeminal nerve (Figure 5A), when mice ingested a hard or soft diet. Ingestion of a hard diet induced a higher expression of Fos-ir cells at the Pr5 than did the ingestion of a soft diet or no diet (Figure 5B, p<0.0005). The sensory information received at the Pr5 is known to be transmitted to the pedunculopontine tegmental nucleus (PTg) via the thalamus [24], the somatosensory cortex [25], and the motor cortex [26]. Ingestion of a hard diet induced remarkable expression of Fos-ir cells at the PTg, while ingestion of a soft diet or no diet did not (Figure 5C, p<0.05). Cholinergic and glutamatergic neurons of the PTg innervate to the substantia nigra pars compacta (SNc) [27], [28], and dopaminergic neurons from the substantia nigra pars compacta innervate to the SVZ [29], [30]. The number of Fos-ir cells at the SNc in mice ingested a hard diet was much greater than that in mice with no diet (p<0.05), while that after ingestion of soft diet was similar to that in mice with no diet (Figure 5D).

It is possible that ingestion of a hard diet induces stronger olfactory sensations than ingestion of a soft diet merely because it takes more time to ingest a hard diet. We explored the excitation of neurons at the OB after ingestion of a hard diet or soft diet. Ingestion of a hard diet induced almost no increase in Fos-ir cells at the OB (data not shown). In aged senescence-accelerated prone (SAMP8) mice, a molarless condition has been shown to induce elevation of the plasma corticosterone concentration [31], [32]. We examined the effects of a hard or soft diet on the plasma corticosterone concentrations under our experimental conditions. The plasma corticosterone concentration in mice fed a soft diet for 3 months was higher than that in mice fed a hard diet but not so significant (data not shown).

## Discussion

The numbers of BrdU-ir cells in the SVZ and OB of adult mice fed the soft diet for 1 month were lower than those of the mice fed the hard diet. The soft diet also affected the adverse responses of mice to butyric acid. However, mice fed the hard diet for 3 months after the soft diet for 1 month showed a level of avoidant behaviors in response to 50% butyric acid similar to that of mice fed only the hard diet. The responses to urinary odor at the OB and Pir were recovered by the hard-diet feeding. The switch to the hard diet for 3 months reversed the decline in neurogenesis at the SVZ in soft-diet-fed mice. Ingestion of a hard diet induced expression of Fos-ir cells at the Pr5 but ingestion of a soft diet did not.

Decreases in neurogenesis at the SVZ impair the various functions related to olfaction. Ablation of adult neurogenesis induced by irradiation of the SVZ does not affect the odor discrimination activity and short-term memory within 30 min, but it does impair long-term odor memory rewarded with water lasting for 30 days [33]. This suggests that a decrease in neurons migrating from the SVZ affects a behavior related to olfactory memory. The blocking of neurogenesis by AraC has been shown to reduce oscillations in the OB [34]. AraC treatment before and during the odor-enrichment period prevents olfactory perceptual learning, which improves odor discrimination [35]. Olfactory perceptual learning is also impaired with aging, which is associated with a reduction of neurogenesis [36]. In the present study, we showed that mice fed a soft diet showed low neurogenesis and did not avoid the odor of 50% butyric acid, while mice fed only a hard diet or a hard diet after a soft one showed normal or recovered neurogenesis and avoided the odor, respectively. These results suggest that the decrease in adult neurogenesis induced by a soft diet impaired the ability of odor cognition for avoidance. Pregnancy stimulates neurogenesis at the SVZ of female mice and this effect is mediated by prolactin, implying that forebrain olfactory neurogenesis may contribute to adaptive behaviors in mating and pregnancy [14]. Neurogenesis is also required for predator and sex-specific responses related to olfaction [37]. Therefore, it is possible that the reduction or stimulation of neurogenesis at the SVZ induces changes in the neural circuits related to aversive responses to odorants in the OB.

The effects of decreases in mastication by the soft-diet feeding or by shortening of the upper molars have been studied in order to explore the effects of mastication on the development of the brain functions of mice after weaning. The level of synaptophysin in the whole cortex [7], and those of brain-derived neurotrophic factor (BDNF) and BrdU-ir cells in the hippocampus [20], [21], of mice fed a soft diet after weaning decrease at the age of 3 months. After 6 months of age, the abilities of the working memory tested with an eight-arm maze [7] and space memory tested with a water maze [38] are reduced in mice fed a soft diet after weaning. In rats, feeding with a soft diet after weaning for 24 weeks reduces BrdU-ir cells in the hippocampus [39]. Rats that undergo extraction of the molar teeth at 7 weeks of age show an impairment of space memory at 24 weeks of age [5]. These results suggest that decreases in mastication reduce brain development in pre-mature animals. In the present study, we did not find a significant change in BrdU-ir cells at the SVZ one month after weaning, because the numbers of BrdU-ir cells varied widely from mouse to mouse (data not shown).

SAMP8 mice have been used as an animal model of old age to explore the effects of impaired mastication on brain functions. The shortening of the upper molars of elderly SAMP8 mice decrease neuron density in the hippocampus CA1, neurogenesis activity in the DG, and spatial learning ability [6], [31], indicating that impaired mastication in old age reduces brain functions related to the hippocampus. In the present study, we showed that soft-diet feeding reduced neurogenesis related to olfactory behaviors at the SVZ and that at the DG in mice in middle age. The present results thus indicate that mastication is also important for brain function in the middle years of life.

There was a discrepancy between the effects at the SVZ and the DG. In general, the neurogenesis activity at the adult SVZ of rodents was vigorous [40] while hippocampal neurogenesis in adult mice is rather small [41]. In the present study, BrdU-ir cells were observed in the SVZ at 1 hour after the injection of BrdU (Figure 1A, B), but BrdU-ir cells were essentially not observed in the DG (data not shown). Feeding of the soft diet for 1 month induced decreases in BrdU-ir cells at the SVZ but not at the DG, suggesting that the effects of impaired mastication appear at the SVZ with vigorous neurogenesis activity earlier than at the DG with poor one. Exercise is known to reduce the age-dependent decline in cell proliferation at the mouse DG [42], [43]. Voluntary running increases the number of new hippocampal granule cells but not the number of adult-generated olfactory granule cells [44]. Long-term running elevates BDNF peptide levels [45], while soft-diet feeding after weaning reduces BDNF levels in the hippocampus [21]. These results suggest that mastication and exercise increase neurogenesis at the DG by increasing BDNF levels. However, BDNF delivered to the lateral ventricle does not increase SVZ neurogenesis in mice, and even decreases neurogenesis in rats [46]. In addition, TrkB is not essential for adult SVZ neurogenesis in mice [46].

At this time, it is unclear how impaired mastication decreases and how hard-diet feeding recovers neurogenesis at the SVZ. In the present study, ingestion of a hard diet induced excitation of neurons at the Pr5. Sensory information from ingestion of the hard diet received at the Pr5 is transmitted to the pedunculopontine tegmental nucleus (PTg) via the thalamus [24], somatosensory cortex [25], and motor cortex [26]. In the present study, ingestion of a hard diet induced remarkable expression of Fos-ir cells at the PTg. Cholinergic and glutamatergic neurons of the PTg innervate to the SNc [27], [28], [47]. In the present work, neurons at the SNc were activated by ingestion of a hard diet (p<0.05). Proliferative precursors in the SVZ express dopamine receptors and receive dopaminergic afferents [29]. Dopamine increases the proliferation of SVZ-derived cells by releasing epidermal growth factor in a PKC-dependent manner in vitro [30]. Dopaminergic neurons in the SNc innervate to the SVZ [29], [30]. Therefore, it is possible that the feeding with a hard diet maintained neurogenesis at the SVZ via the Pr5, PTg and SNc.

In aged mice, removal of the upper molars increases plasma corticosterone levels [31]. In this study, the plasma corticosterone concentrations of middle-aged mice fed a soft diet were slightly higher than those of mice fed a hard diet. An increase in plasma corticosterone induced by chronic stress has been shown to decrease the number of neural stem cells in the mouse SVZ [48]. These data suggest that changes in mastication conditions through the ingestion of a soft diet may also decrease adult neurogenesis at the SVZ via corticosterone.

## Materials and Methods

All experiments were carried out in accordance with the Guidelines for the Use of Laboratory Animals of the Asahikawa Medical University and approved by the Committee of Asahikawa Medical College for Laboratory Animal Care and Use (approval ID: 11014).

### Animals

A total of 131 C57BL/6 female mice (from 24 to 28 weeks old) were used. Five mice were fed 25 g of hard or soft diet per week in the same cage. The mice and the hard and soft diets were obtained from Sankyo Laboratory Co. (Sapporo, Japan); both diets had the same nutritional composition. The relative body weights of the mice fed the soft diet for 1 month were essentially the same as those of the mice fed the hard diet (data not shown). To explore the reversibility of soft-diet feeding, mice were switched to a hard diet for 1 or 3 months after having been fed the soft diet for 1 month. The relative body weights of these mice were also essentially the same as those of the mice fed a hard diet or soft diet (data not shown). The mice were injected intraperitoneally with BrdU (50 mg/g; Sigma, St. Louis, MO), a marker of DNA synthesis, at 1 h before exsanguinating to observe BrdU-ir cells at the SVZ. During each of the 3 days from 1 week before killing, the mice were injected with two shots of BrdU per day to observe BrdU-ir cells at the OB and DG.

### Stimulation with urinary odor or diet

For odor stimulation, urine was collected from 10 males using a metabolic cage. Five milliliters of a urine mixture taken from 10 males was sprayed on the soiled bed made of paper (SLC, Hamamatsu, Japan) in the cage. Before stimulation with hard or soft diet, mice were starved for 2 days. The animals were deeply anesthetized with pentobarbital sodium (35 mg/kg) 90 min after stimulation with urinary odor or diet.

### Tissue processing and BrdU and Fos immunohistochemistry

After deep anesthetization with pentobarbital sodium (35 mg/kg), the BrdU-injected mice were exsanguinated by perfusion through the heart with phosphate-buffered saline (PBS), then fixed with 4% paraformaldehyde. The brain was removed and cut sagittally on a vibratome at a thickness of 100 µm. For the detection of BrdU-labeled nuclei, sections were first incubated in 2 N HCl for 30 min at 37°C and rinsed in 0.1 M boric acid (pH 8.0) for 5 min, followed by washing in PBS with 0.4% Triton X-100 (PBSx). The sections were then incubated in PBSx with 0.6% H_2_O_2_ for 15 min, followed by washing in PBSx. After 1 h of incubation in 3% normal goat serum, the sections were incubated with anti-BrdU monoclonal antibody (1∶400; Roche Diagnostics, Mannheim, Germany) in PBSx for 24 h at room temperature. The sections were then rinsed in PBSx and incubated with biotinylated goat anti-mouse IgG (1∶100; Vector Laboratories, Burlingame, CA) for 1 h. The sections were rinsed again in PBSx, incubated with ABC (ABC Elite kit; Vector Laboratories) for 1 h, and developed with DAB/H_2_O_2_ (0.05% DAB and 0.003% H_2_O_2_ in 0.05 M Tris-HCl buffer) for 5 min.

For the detection of c-Fos immunoreactivity, the sections were first treated with 0.6% H_2_O_2_ for 15 min in PBSx, followed by two washes with PBSx. After 1 h incubation in 3% normal goat serum, the sections were incubated overnight at room temperature with c-Fos polyclonal antibody (1∶8000, Ab-5; Calbiochem, La Jolla, CA) in PBSx. All sections treated in this manner were then rinsed with PBSx and incubated with biotinylated goat anti-rabbit IgG (1∶200; Vector Laboratories) for 1 h. The sections were rinsed again in PBSx, incubated with ABC (ABC Elite kit; Vector) for 1 h, and developed with DAB/H_2_O_2_ for 12 min.

The sections were rinsed with water, mounted, and dehydrated before being covered with cover slips. The thickness of dehydrated slices was 20.4+2.0 µm (n = 10). Images of BrdU-ir cells at the SVZ and DG were captured by a CCD camera (DP72; Olympus, Tokyo, Japan) attached to an inverted microscope (BX51; Olympus) with a 10× objective lens (UPlan FLN, 10x/0.30; Olympus), and displayed on the monitor. Then, BrdU-ir cells in the rostral wall of the SVZ and in the DG were counted by naked eye. At the OB, BrdU-ir and Fos-ir cells in a rectangle area (600 µm × 450 µm) near the AOB were similarly counted by using 20× objective lens (UMPlan FI, 20×/0.46; Olympus). At the Pir, Pr5, PPN and SNc, Fos-ir cells were photographed with 5× objective lens (MPlane N, 5x/0.10; Olympus), and counted by the naked eye. The brain nuclei borders were distinguished by overlaying a brain atlas drawing adapted from the stereotaxic atlas of the mouse brain [49] on our photographs. All BrdU-ir cells or Fos-ir cells in dehydrated sections were visualized in the same or nearly the same (20× objective lens) focal plane in our experimental equipment.

### Double-staining immunohistochemistry

Doublecortin (DCX) is one of the suitable markers for adult neurogenesis [50]. For double labeling with BrdU and DCX, sections were cut at a thickness of 40 µm. After pretreatment with 2 N HCl, the sections were incubated overnight at 4°C in anti-BrdU (1∶100; Roche Diagnostics) and rabbit anti-DCX antibodies (1∶300; Cell Signaling Technology, Danvers, MA) and then incubated for 1 h at room temperature in Alexa Fluor 488 goat anti-mouse IgG and Alexa Fluor 594 goat anti-rabbit IgG (1∶2,000; Invitrogen, Carlsbad, CA). The sections were then mounted with VECTASHIELD Mounting Medium (Vector Laboratories Inc., Burlingame, CA). Individual double-labeled cells in the SVZ were visualized using a confocal fluorescence microscope (Fluoview FV1000-D; Olympus, Tokyo, Japan).

### Avoidance of butyric acid

Odor preferences of individual female mice were tested in a Plexiglas Y-maze preference apparatus with 45 cm arms according to the methods described in previous studies [51]–[53]. A 10 µl aliquot of 50% butyric acid or a control stimulus (water) absorbed in filter paper in a petri dish was placed at the end of each arm (40 cm) of the Y, and the start box was placed at the end of the long arm (45 cm). Clean air was supplied from the end of each short arm, where 50% butyric acid or water was placed, to the end of the long arm, where the mice were initially placed. A mouse was placed in the starting position and then the barrier was removed to expose the odor stimuli. During the subsequent 4 min, the length of time each mouse spent in each arm was recorded. Preference ratio was defined as the duration of time a mouse spent in the arm with odor stimuli divided by the total time spent in the two arms combined.

### Blood sampling and corticosterone assay

Plasma sampling was performed between 20:00 and 21:00. Blood samples were collected from the hearts into chilled tubes and then centrifuged at 750×g for 30 min at 4°C. The supernatants were stored at −80°C until assay. Corticosterone levels were determined using an EIA kit (Yanaihara Institute Inc., Shizuoka, Japan) according to the manufacturer's instructions. The measurement was performed in duplicate.

### Statistical analysis

The numbers of BrdU-ir cells, numbers of Fos-ir cells, preference ratio and plasma corticosterone concentration were compared by the U-Mann Whitney test or analysis of variance (ANOVA) with Fisher's PLSD post-hoc testing. Statistical analyses were performed with StatView version 5.0 (SAS Institute, Cary, NC). Data are expressed as the mean ± SEM.

## Supporting Information

Figure S1 BrdU-ir cells in the DG of mice fed a hard or soft diet. Sagittal sections of the DG of mice fed a hard diet (A and C) or a soft diet (B and D) for 1 or 3 months, respectively. Scale bar: 200 µm. E and F: The numbers of BrdU-ir cells in 1 mm thickness from the lateral 0.84 mm section of the DG to the lateral side. Black and white columns indicate the numbers of BrdU-ir cells in the DG of mice fed the hard diet and the soft diet for 1 month (E) or 3 months (F), respectively. The data from each group were cast into a two-factor ANOVA as follows: hard or soft and period. The main effect of diet was found to be significant (F(1, 156) = 5.573, p<0.05). n = 5 (both columns in E and the black column in F). n = 4 (white column in F). *: p<0.05.
(TIF)

Figure S2 Confocal micrographs of DCX/BrdU double-immunolabeled signals in the SVZ of mice fed a hard diet for 3 months after being fed a soft diet. Sagittal sections of the SVZ of mice fed only a hard diet (A), only a soft diet (B), or a hard diet for 3 months after a soft diet for 1 month (C). Green and red indicate BrdU- and DCX-immunoreactivities, respectively. Scale bar: 40 µm. D: The average numbers of BrdU-ir signals surrounded by DCX-ir signals as double-labeled cells in the SVZ of mice fed only the hard diet (black column), only the soft diet (white column), or the hard diet for 3 months after the soft diet for 1 month (gray column). The quantification was performed using each four serial sections of 40 µm thickness from Figure 110 of the mouse atlas (lateral 1.08 mm) to the lateral side. In each section, the double-labeled cells were counted on a 2 µm-thick optical slice having the largest number of BrdU-ir signals. Individual BrdU-ir signals were overlapped with DAPI signals (not shown). The average number of signals in four slices in one animal was analyzed. n = 3 for the black and white columns, n = 4 for the gray column. *: p<0.05.
(TIF)

Figure S3 Fos-ir cells in the Pr5 of mice after in ingestion of the hard or soft diet. Sagittal sections of the Pr5 of mice after ingestion of the hard diet (A), soft diet (B), and no diet (C), respectively. Scale bar: 500 µm.
(TIF)
