# Supplementary figures and images for: Hard-Diet Feeding Recovers Neurogenesis in the Subventricular Zone and Olfactory Functions of Mice Impaired by Soft-Diet Feeding
Source: PLoS One. 2014 May 9;9(5):e97309. doi: 10.1371/journal.pone.0097309 (PMC4016307; doi:10.1371/journal.pone.0097309)

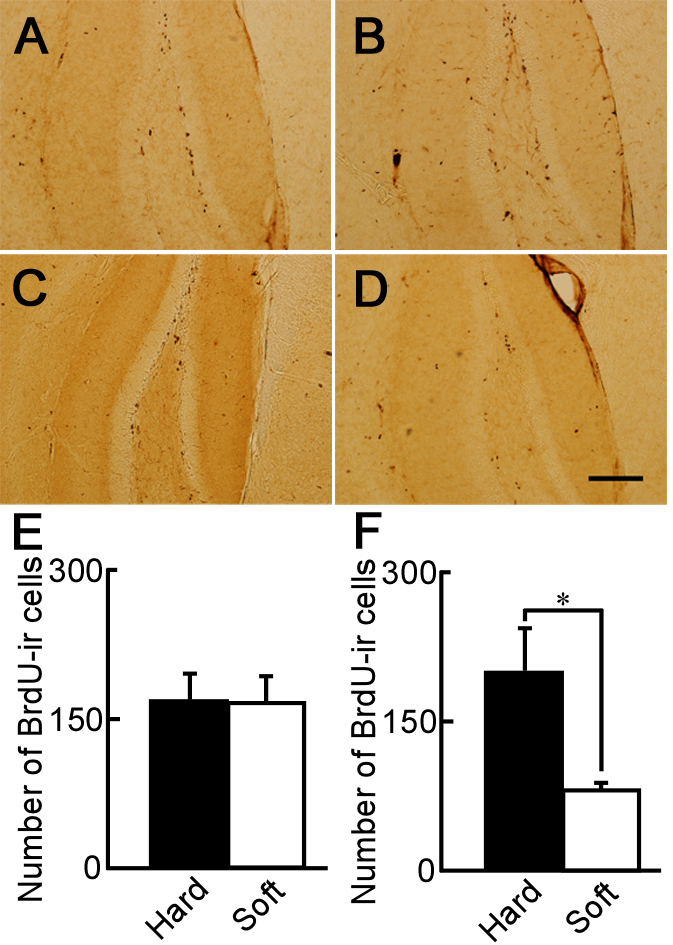

Supplement: Figure S1 — BrdU-ir cells in the DG of mice fed a hard or soft diet. Sagittal sections of the DG of mice fed a hard diet (A and C) or a soft diet (B and D) for 1 or 3 months, respectively. Scale bar: 200 µm. E and F: The numbers of BrdU-ir cells in 1 mm thickness from the lateral 0.84 mm section of the DG to the lateral side. Black and white columns indicate the numbers of BrdU-ir cells in the DG of mice fed the hard diet and the soft diet for 1 month (E) or 3 months (F), respectively. The data from each group were cast into a two-factor ANOVA as follows: hard or soft and period. The main effect of diet was found to be significant (F(1, 156) = 5.573, p<0.05). n = 5 (both columns in E and the black column in F). n = 4 (white column in F). *: p<0.05. (TIF) [file pone.0097309.s001.tif]

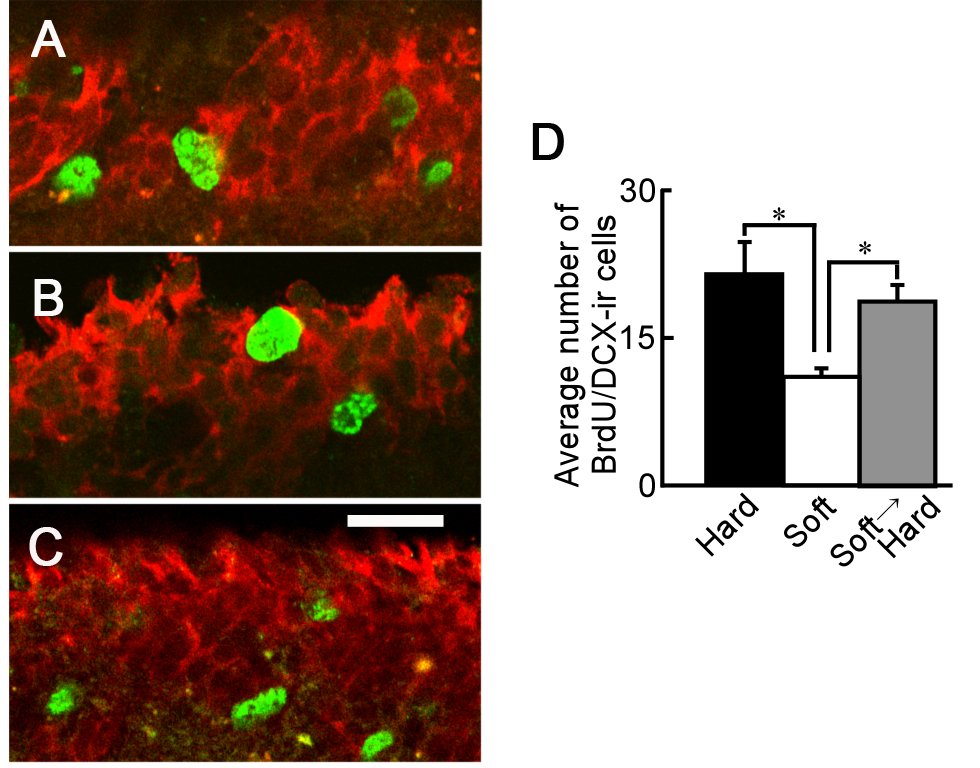

Supplement: Figure S2 — Confocal micrographs of DCX/BrdU double-immunolabeled signals in the SVZ of mice fed a hard diet for 3 months after being fed a soft diet. Sagittal sections of the SVZ of mice fed only a hard diet (A), only a soft diet (B), or a hard diet for 3 months after a soft diet for 1 month (C). Green and red indicate BrdU- and DCX-immunoreactivities, respectively. Scale bar: 40 µm. D: The average numbers of BrdU-ir signals surrounded by DCX-ir signals as double-labeled cells in the SVZ of mice fed only the hard diet (black column), only the soft diet (white column), or the hard diet for 3 months after the soft diet for 1 month (gray column). The quantification was performed using each four serial sections of 40 µm thickness from Figure 110 of the mouse atlas (lateral 1.08 mm) to the lateral side. In each section, the double-labeled cells were counted on a 2 µm-thick optical slice having the largest number of BrdU-ir signals. Individual BrdU-ir signals were overlapped with DAPI signals (not shown). The average number of signals in four slices in one animal was analyzed. n = 3 for the black and white columns, n = 4 for the gray column. *: p<0.05. (TIF) [file pone.0097309.s002.tif]

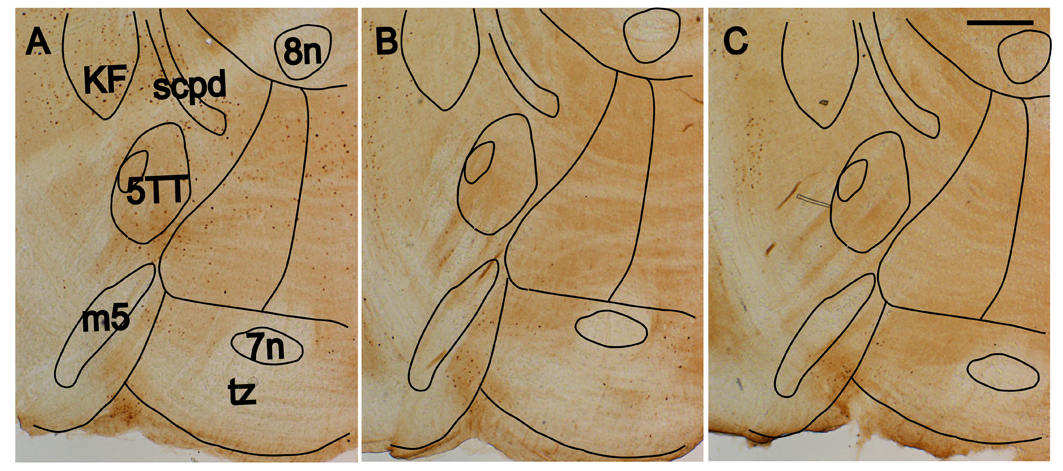

Supplement: Figure S3 — Fos-ir cells in the Pr5 of mice after in ingestion of the hard or soft diet. Sagittal sections of the Pr5 of mice after ingestion of the hard diet (A), soft diet (B), and no diet (C), respectively. Scale bar: 500 µm. (TIF) [file pone.0097309.s003.tif]
